# Stability of Nε-Carboxymethyllysine and Nε-Carboxyethyllysine in Canine Urine Under Extended Room Temperature Storage

**DOI:** 10.3390/ani16060917

**Published:** 2026-03-14

**Authors:** Nicole Renée Cammack, Stephanie Archer-Hartmann, Bhoj Kumar, Christian Heiss, Parastoo Azadi, Joseph Bartges

**Affiliations:** 1 Department of Small Animal Medicine & Surgery, College of Veterinary Medicine, University of Georgia, Athens, GA 30602, USA; jbartges@uga.edu; 2 Complex Carbohydrate Research Center, University of Georgia, Athens, GA 30602, USA; sarcher@ccrc.uga.edu (S.A.-H.); bhoj.kumar@uga.edu (B.K.); cheiss@ccrc.uga.edu (C.H.); azadi@ccrc.uga.edu (P.A.)

**Keywords:** advanced glycation end products—AGEs, canine nutrition, dog urine stability, urinary AGE stability, urinary metabolomics

## Abstract

Advanced glycation end products (AGEs) such as Nε-carboxymethyllysine (CML) and Nε-carboxyethyllysine (CEL) are implicated in chronic disease processes in humans and may serve as biomarkers of dietary exposure and metabolic health. Urinary measurement of AGEs is of interest due to its non-invasive nature and relevance to biobanking and field-based sample collection; however, AGE stability in urine under common handling conditions has been poorly characterized. This study evaluates the short-term stability of CML and CEL in canine urine stored at room temperature (20 °C) for up to 168 h prior to −80 °C storage. Midstream free-catch urine samples from eight healthy dogs were aliquoted, stored at defined intervals, and analyzed in duplicate using liquid chromatography–mass spectrometry (LC-MS) with isotope-labeled standards. Results demonstrate minimal detectable changes in CML and CEL concentrations, as well as in the CML/CEL ratio, over the ambient storage period. Inter-replicate agreement is high, and regression and non-parametric analyses show no association between storage duration and analyte concentration. These findings indicate that urinary CML and CEL measurements may remain reliable despite delayed processing, supporting field-based sampling and retrospective analyses. Evaluation of additional AGE species and storage conditions will further inform best practices for sample handling in veterinary and comparative biomedical research.

## Simple Summary

Advanced glycation end products are compounds that form in the body and in food during heating and processing. They have been linked to chronic diseases in people and may also play a role in dog health. Measuring these compounds in urine is appealing because urine collection is non-invasive and useful for nutrition and health studies. However, urine samples are not always frozen immediately after collection, and it is unclear whether delays in processing affect the accuracy of these measurements. In this study, urine was collected from healthy dogs and stored at room temperature for up to seven days before freezing. Two advanced glycation end products were measured using a highly sensitive laboratory method. The results showed that concentrations of these compounds remained stable over time, even when freezing was delayed. This suggests that short-term room temperature storage does not substantially affect their measurement in dog urine. These findings support the use of urine samples collected under real-world conditions and provide practical guidance for researchers conducting nutrition, metabolism, and health studies in dogs.

## 1. Introduction

There is growing interest in the role of AGEs in health and disease in humans and dogs [1,2,3]. AGEs result from non-enzymatic reactions between reducing sugars and proteins [4]. Reactions occur endogenously during normal metabolism and exogenously during thermal processing of dietary ingredients [5]. Over 20 AGEs have been found within human biofluids, tissues and in food products, and are commonly categorized into groups based on chemical structure and fluorescence properties [5,6]. Dietary AGEs are the main source of the AGE body pool in addition to endogenous AGE production, and elimination of AGEs is done via gastrointestinal and urinary excretion [7]. An estimated one-third of gastrointestinal-absorbed AGEs are excreted in urine within 48 h in healthy humans [8], although excretion may be lower with renal disease.

In humans, consumption of ultra-processed diets with high amounts of AGEs is associated with chronic diseases such as diabetes mellitus, arthritis, cancer, chronic kidney disease, and cognitive dysfunction [9]. Dogs share similar chronic diseases and environmental exposures, notably consumption of ultra-processed diets. Commercial pet foods contain similar levels of AGEs found in ultra-processed human foods, although dogs consuming commercial diets consume 122 times more AGEs than humans consuming a Western diet on a metabolic body weight basis [10]. Higher intake is attributed to dogs being fed the same diet (e.g., kibble, canned) often for a lifetime with little to no variety. Additional studies demonstrate that dogs consuming high-AGE-containing diets may serve as translational models [1,2].

AGEs may be measured in diet, serum, and urine [11]. Biobanking initiatives of samples such as urine are becoming more frequent [12,13,14,15]. Different metabolite classes exhibit varying sensitivity to storage conditions such as time, temperature fluctuations, light, and oxidation [16,17,18]. As such, time delays, temperature fluctuation, and contamination prior to storage and data acquisition may result in metabolite instability in biosamples [19].

While AGEs in food products are generally considered stable over time, urinary stability of these compounds is poorly characterized. Lack of standardized collection and storage protocols can affect sample stability and result in inaccurate estimation of AGE concentrations [19]. CML and CEL are widely studied nonfluorescent, non-cross-linked advanced glycation end products [5]. CML arises from multiple pathways, including oxidation of Amadori products, glyoxal-mediated reactions from carbohydrate or lipid oxidation, and free radical processes [20]. CEL is primarily formed through the reaction of lysine residues with methylglyoxal or triose-phosphate, both reactive dicarbonyl compounds generated during lipid peroxidation and sugar degradation [21]. Both are considered important biomarkers for assessing AGE exposure and the endogenous AGE pool and have been associated with numerous conditions in humans [22,23]. The formation of these and other AGEs is modulated by temperature, time, moisture content, and the presence of reactive substrates such as sugars, lipids, and proteins, as well as inhibitory compounds [4]. Although their roles in canine health are not defined, AGEs are believed to contribute to chronic diseases in dogs, similarly to humans, and their presence and relationship to diet have been investigated in canine urine [2,7].

The lack of stability data on AGEs limits confidence in cross-study comparisons and potentially undermines data interpretation in large-scale or retrospective research. Stability of CML and CEL has been evaluated in plasma and serum where collection tube type, delayed processing, storage temperature, and multiple freeze–thaw cycles do not significantly alter measured concentrations [24]. CML stability has been investigated in food systems, demonstrating storage conditions significantly influence AGE accumulation. Specifically, CML levels in skim milk powder increased substantially during prolonged storage under higher humidity and temperature, highlighting AGEs can continue to form post-processing if conditions are unfavorable [25]. The disparity of AGE stability between foods and biofluid demonstrates the need for validation studies assessing the stability of CML and CEL in urine. Furthermore, factors such as cellular debris and bacterial contamination may contribute to sample instability in urine [26,27,28,29,30], demonstrating a need for standardized processing protocols in AGE research.

This study evaluates the short-term stability of CML, CEL, and the CML/CEL ratio in canine urine under routine handling conditions, with samples held at 20 °C for up to 168 h before −80 °C storage. This time frame reflects realistic delays that commonly occur between sample collection and analysis, particularly in field-based studies or when samples are shipped to centralized laboratories or biobanks. We hypothesized that concentrations of CML, CEL, and the CML/CEL ratio would exhibit minimal change during storage at 20 °C prior to −80 °C freezing.

## 2. Materials and Methods

### 2.1. Sample Collection, Processing and Storage

Eight client-owned dogs (age range 1–15 years; 7 neutered males and 1 spayed female) were included. Breeds comprised a Dachshund/Labrador Retriever mix (*n* = 1), a Labrador Retriever mix (*n* = 1), Pit Bull-type mixes (*n* = 2), a Golden Retriever (*n* = 1), and Goldendoodles (*n* = 3). Because urinary metabolite profiles can vary by biological sex, sex and neuter status were recorded for all dogs and considered during interpretation; however, the sample size limited formal assessment of sex effects.

**Figure 1:**
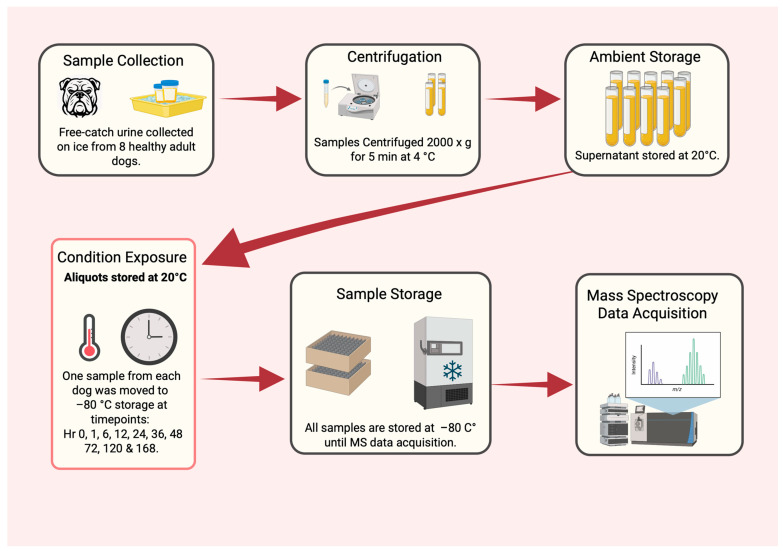
Overview of dog urine collection, handling and storage. Created in https://BioRender.com.

All urine samples were collected as first-morning voids via midstream free-catch urine samples (90–120 mL) using sterile 120 mL polypropylene specimen containers (Dynarex, Orangeburg, NY, USA, item #4353) by trained personnel. Immediately after collection, samples were placed on ice and transported to the laboratory within 10 min. Upon arrival, samples were vortexed for 30 s (VWR Vortex Mixer; VWR International, Radnor, PA, USA), and 1 mL aliquots were transferred into polypropylene microcentrifuge tubes (Eppendorf Flex-Tube; Eppendorf, Hamburg, Germany; item #022363531) and maintained on ice. Each sample was divided into eleven duplicate aliquots corresponding to storage time points of 0, 1, 3, 6, 12, 24, 36, 48, 72, 120, and 168 h. Aliquots were centrifuged at 2000× *g* for 5 min at 4 °C (Eppendorf 5417 C), after which supernatants were transferred into sterile 2 mL cryovials. Aliquots designated as hour 0 were immediately frozen at −80 °C, while the remaining samples were stored at 20 °C and transferred to −80 °C at their respective time points. An overview of sample collection, processing, and storage is provided in Figure 1. All procedures were conducted in accordance with applicable animal welfare legislation and were approved by The University of Georgia’s Clinical Trials Committee, which is also referenced in the Ethics statement.

### 2.2. Reagents and Standards

Formic acid (≥99% purity) (Thermo Fisher Scientific, Waltham, MA, USA), acetonitrile (ACN) (Thermo Fisher Scientific, Waltham, MA, USA), and distilled water were all LC-MS grade (Thermo Fisher Scientific, Waltham, MA, USA). Internal standards were Nε-(1-carboxymethyl)-L-lysine-d_3_ (CML-d_3_, Cayman Chemical, Ann Arbor, MI, USA, Item#: 26785) and Nε-(1-carboxyethyl)-L-lysine-d_4_ (CEL-d_4_, Iris Biotech, Marktredwitz, Germany, Product Code: HAA2941), each prepared at 10 µM in 0.1% formic acid.

### 2.3. Mass Spectrometry Methodology

Quantification of CML and CEL was performed as previously described by Bridglalsingh et al. (2024), with minor modifications [2]. A 0.5 mL aliquot of urine was filtered using a 10 kDa molecular weight cutoff centrifugal filter (Pall Corporation, Singapore, Singapore). The filtrate was diluted 50-fold into a solution containing 10 µM CML-d_3_ and 10 µM CEL-d_4_ in 0.1% formic acid. Analyses were conducted using an Orbitrap Q Exactive mass spectrometer (Thermo Fisher Scientific) coupled to a Vanquish UPLC system (Thermo Fisher Scientific) equipped with an electrospray ionization source operating in positive ion mode. Chromatographic separation was achieved on a SeQuant^®^ ZIC-HILIC column (3.5 µm, 150 × 2.1 mm) using a stepwise solvent gradient. Solvent A consisted of LC–MS grade water with 0.1% formic acid, and solvent B consisted of LC–MS grade acetonitrile with 0.1% formic acid. The flow rate was 350 µL/min, with the following elution profile: 0–2 min, 90% B; 2–3 min, linear gradient to 65% B; 3–5 min, 65% B; 5–7 min, linear gradient to 40% B; 7–10 min, 40% B; 10–12 min, linear gradient to 90% B; and 12–28 min, 90% B. Samples were injected at a volume of 1 µL. Ion detection was performed using selected ion monitoring with *m*/*z* (mass-to-charge ratio) values of 205/208 for CML/CML-d_3_ and 219/223 for CEL/CEL-d_4_. Data were acquired using Thermo Fisher Excalibur software (version 4.6), and quantification was based on the ratio of extracted ion peak areas of the internal standards to the corresponding endogenous AGE peaks.

Analytical reproducibility was assessed by analyzing all samples twice (N1 and N2) under identical LC–MS conditions, with samples maintained in a thermostated autosampler at 10 °C. Replicate analyses were conducted on separate days using the same instrumentation, columns, mobile phases, and analytical protocol. Both runs were included in downstream statistical analyses to evaluate inter-assay agreement and temporal stability of CML and CEL concentrations. Raw data from both analytical runs are provided in Supplementary Materials.

### 2.4. Data Analysis

To assess inter-replicate variability for CML and CEL, concentrations from duplicate analytical runs (*n* = 176 total observations; 88 per replicate) were compared using descriptive statistics and the Kruskal–Wallis test, as values were non-normally distributed based on Shapiro–Wilk tests of normality. Boxplots were used to visualize inter-replicate distributions across all dogs. Aggregated CML and CEL concentrations (*n* = 176 per analyte) were further evaluated using histograms with overlaid kernel density estimates to characterize distributional properties and inform selection of statistical tests. CML/CEL ratios were calculated and visualized using the same approach.

To evaluate the influence of room temperature storage duration on AGE concentrations, data from both analytical replicates were included in linear regression models for CML, CEL, and the CML/CEL ratio, with storage time (hours) as the independent variable. The magnitude of temporal change was assessed by examination of regression slopes and corresponding confidence intervals, with the model fit evaluated using R^2^ values and ANOVA. Residuals were assessed for normality and homoscedasticity using Shapiro–Wilk tests and residual diagnostic plots. Non-parametric analyses were additionally performed to examine changes across discrete time points, with medians visualized using color-coded boxplots by dog. Kruskal–Wallis tests were used to assess differences in central tendency, followed by Steel–Dwass–Critchlow–Fligner post hoc comparisons to account for multiple testing.

For interpretation of analytical stability, we used standard bioanalytical method validation criteria [31]. Concentrations within ±15% of the nominal value (±20% at the lower limit of quantification) were considered acceptable for accuracy, precision, and stability. Temporal changes in CML and CEL over 168 h were then evaluated against these predefined limits.

Normalization to creatinine or specific gravity was not applied, as the objective of this study was to assess absolute analyte stability under controlled pre-analytical conditions, independent of physiological dilution effects. This approach is consistent with the study’s pre-analytical focus, in which within-sample temporal stability was the primary endpoint. It is worth noting that creatinine normalization has recognized limitations in urinary metabolomics, including its dependence on renal function, muscle mass, hydration status, and diet, which can introduce systematic bias when used as a sole reference marker [32,33]. Current metabolomics literature increasingly favors alternative approaches such as probabilistic quotient normalization, osmolality adjustment, or total useful signal normalization over creatinine adjustment for studies where biological interpretation across individuals is required [34].

## 3. Results

Comparison between N1 and N2 indicates high reproducibility for CML and CEL quantifications. Medians concentrations of 9.13 µg/mL interquartile range (IQR): 4.65–33.46) and 7.00 µg/mL (IQR: 4.33–28.29) for CML, and 2.36 µg/mL (IQR: 1.28–5.95) and 1.86 µg/mL (IQR: 1.20–5.49) for CEL were observed. Concentrations were highest in the same dogs across both replicates for each AGE (CML: 98.81 vs. 66.74 µg/mL; CEL: 17.07 vs. 10.76 µg/mL). Kruskal–Wallis testing found no statistically significant differences between replicates (CML: H = 1.52, *p* = 0.218; CEL: H = 1.29, *p* = 0.256), indicating consistent analytical performance.

**Figure 2:**
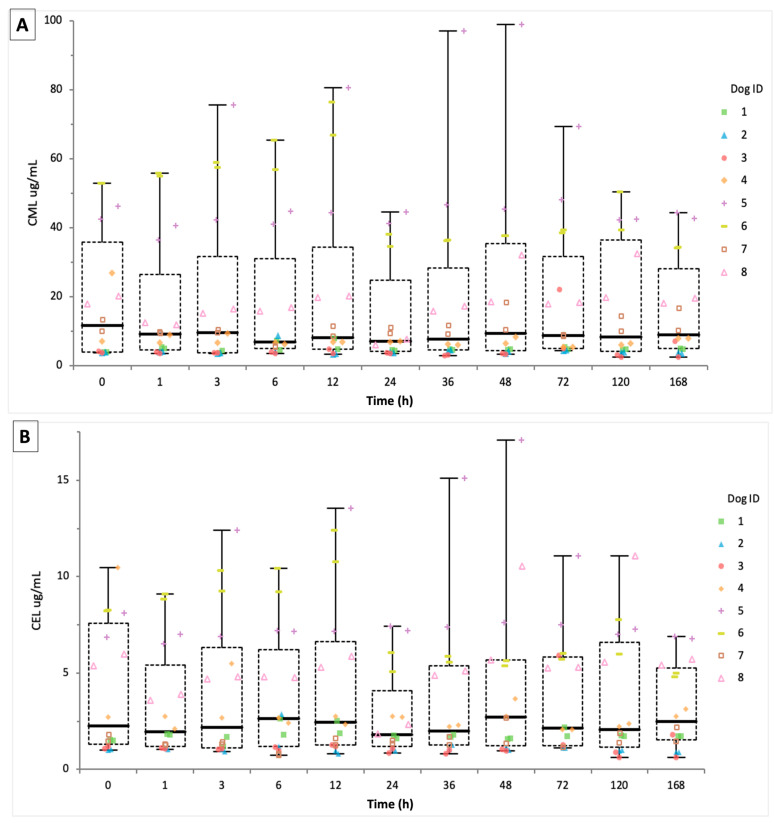

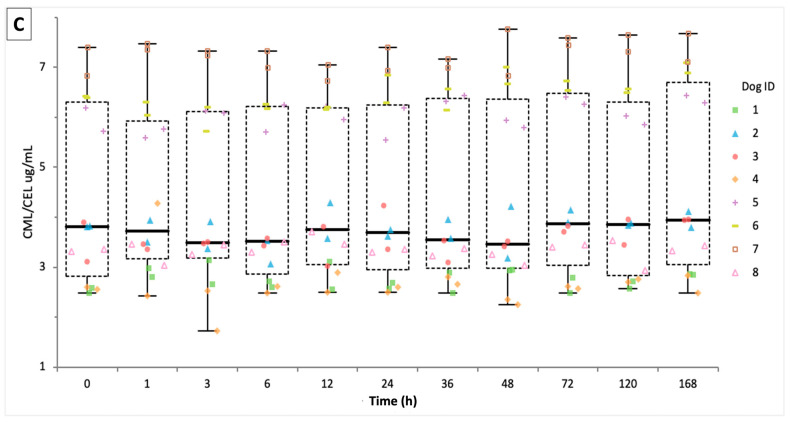
Temporal changes in CML, CEL, and the CML/CEL ratio in canine urine stored at room temperature over 168 h. Boxplots display concentrations of (**A**) Nε-carboxymethyllysine (CML), (**B**) Nε-carboxyethyllysine (CEL), and (**C**) the CML/CEL ratio across eleven time points (0, 1, 3, 6, 12, 24, 36, 48, 72, 120, and 168 h). Data represent combined technical replicates (*n* = 176 per analyte). Individual dogs are color-coded to illustrate inter-individual variability, and horizontal black lines indicate group medians. Across all analytes, median values showed minimal variation over time, with Kruskal–Wallis tests indicating no statistically significant differences across time points (CML: *p* = 0.9991; CEL: *p* = 0.9993; CML/CEL ratio: *p* = 0.9993). These results indicate that CML, CEL, and the CML/CEL ratio exhibited minimal observable change within urine of individual dogs under ambient storage conditions for up to seven days.

Boxplots displayed overlapping distributions with consistent interquartile ranges (Figure 2A,B). CML aggregate values (*n* = 176) were positive and concentrated between 2 and 20 µg/mL with a peak near 10 µg/mL and outliers >50 µg/mL in the upper range of the plot. Additionally, CEL values were also positively skewed, with majority <2 µg/mL and a maximum near 16 µg/mL. Histograms for each AGE deviated from normality, suggesting inter-individual variability and temporal heterogeneity.

Linear regression analysis revealed minimal changes in urinary concentrations over time for both AGEs. The slope was −0.0138 µg/mL/h (95% CI: −0.0727 to 0.0451, *p* = 0.6447), intercept 23.51 µg/mL (95% CI: 19.46 to 27.57), and adjusted R^2^ = 0.001 for CML. For CEL, the slope was −0.00236 µg/mL/h (95% CI: −0.0114 to 0.0072, *p* = 0.6269), intercept 4.60 µg/mL (95% CI: 3.94 to 5.26), and adjusted R^2^ = −0.004. Additionally, F-tests (CML: F = 0.21; CEL: F = 0.24) indicate that time did not explain meaningful variance in AGE concentrations. Residual plots did not show heteroscedasticity or autocorrelation. Lastly, Shapiro–Wilk testing confirmed non-normality (CML: W = 0.76, *p* < 0.0001; CEL: W = 0.87, *p* < 0.0001).

For the CML/CEL ratio, linear regression analysis showed minimal change over the storage period. The slope was 0.001638 (95% CI: −0.003449 to 0.006725; *p* = 0.5259), intercept of 3.60 µg/mL (95% CI: 3.37 to 3.82) and adjusted R^2^ = 0.002. The F-test (F = 0.40) indicated that time did not explain meaningful variance in the CML/CEL ratio. Residual plots showed no heteroscedasticity or autocorrelation, with Shapiro–Wilk also confirming non-normality (W = 0.90, *p* < 0.0001) for the CML/CEL ratio (Figure 2C).

## 4. Discussion

Our study demonstrates that urinary concentrations of CML and CEL exhibited minimal detectable change within urine of individual dogs under ambient storage conditions for up to seven days prior to storage at −80 °C. Observed changes in concentrations over time were small and within the range of analytical variability. This suggests these analytes may remain relatively stable during common sample handling delays. These findings provide baseline data to inform initial sample handling protocols in canine urine AGE research, supporting more consistent and reliable biomarker analyses across field studies and biobanking efforts.

Characterizing short-term stability of AGEs in urine is important for expanding biobanking capability and ensuring sample reliability under realistic collection and transport conditions. In large-scale studies and multisite collaborations, immediate access to −80 °C storage is often impractical. The minimal changes observed over 168 h suggest that urinary AGE measurements may remain reliable despite logistical delays, including mailing, temporary refrigeration, or ambient storage prior to processing. This improves the feasibility of longitudinal studies, enhances confidence in archived samples, and supports retrospective analysis where urinary AGE profiles are of interest.

To the authors’ knowledge, stability of AGEs in urine has not been previously investigated. Existing studies have focused primarily on plasma or serum, where delayed processing and freeze–thaw cycles appear to exert minimal influence on CML and CEL concentrations. However, plasma and urine differ substantially in matrix composition and susceptibility to degradation pathways [35]. The present findings therefore provide urine-specific validation that supports the use of urinary AGEs in dietary and disease-related investigations within comparative biomedical research.

The strengths of this study include modeling realistic handling conditions, use of MS with isotope-labeled standards, and inclusion of multiple time points extending to 168 h. Several limitations should also be acknowledged. Only two AGE species were evaluated, storage was assessed under a single controlled temperature condition, and sampling was restricted to healthy adult dogs. Results may not extrapolate directly to diseased populations or samples exposed to greater environmental variability. In addition, while no statistically significant changes were detected over time, the study is not specifically powered to demonstrate equivalence or stability within predefined acceptance limits. Inter-individual variability in CML and CEL concentrations exceeded temporal variability, highlighting the need for future studies to establish reference ranges. Further work should also extend stability assessments to additional AGEs, including argpyridamine, methylglyoxal-derived hydroimidazolone (MG-H1), pyrraline, pentosidine, and related compounds, to develop a more comprehensive understanding of AGE behavior in urine. These findings address pre-analytical stability under controlled conditions and do not assess biological variability, dietary exposure effects, or disease-related differences in urinary AGE concentrations.

Bacterial contamination originating from the distal urogenital tract has been shown to modify urinary metabolites through metabolic activity, degradation processes and pH shifts [26,28,29,36,37,38]. Cellular debris can alter metabolomic profiles through enzymatic activity and pH shifts [26]. These are well-known challenges in urinary metabolomics and related biomarker investigations. Improper handling during collection or processing may exacerbate these effects, potentially compromising sample integrity and stability [28,30]. In the present study, we conducted gentle centrifugation to remove cellular debris without inducing cellular breakage, thereby reducing risks of pH alteration, enzymatic degradation, and oxidative processes that may influence metabolite stability.

Although filtration was not performed and low-level bacterial contamination cannot be excluded, the chemical robustness of CML and CEL suggests these factors likely exerted minimal influence on measured concentrations. CML and CEL are widely regarded as chemically stable AGEs and are routinely used as marker compounds in foods and biological samples [5,39]. Bacterial degradation of CML has been demonstrated only under specific high-anaerobic conditions at body temperature using enriched growth media with specialized intestinal bacteria [40]. These conditions are distinctly different from the aerobic, ambient-temperature storage evaluated in the present study. Although comparable microbial degradation pathways have not been reported for CEL, the minimal temporal change observed in CML and CEL concentrations over 168 h in the present study suggests that incidental uropathogens introduced during free-catch collection exert little measurable impact on these analytes under routine urine-handling conditions.

Future studies targeting broader or more labile metabolite classes may benefit from incorporating additional pre-analytical controls to further enhance sample integrity. Broader classes of metabolites relevant to oxidative stress, inflammation, and metabolic health warrant similar evaluation in this context. Additional temperature conditions (e.g., uncontrolled fluctuations in temperature during mailing, storage on ice, and refrigeration) should be systematically evaluated to guide more robust pre-analytical recommendations for veterinary and human biobanking initiatives.

Together, these findings provide practical, urine-specific guidance for handling and storage of AGE biomarkers in canine nutrition, metabolomics, and comparative biomedical research.

## 5. Conclusions

This study demonstrates that urinary CML and CEL concentrations remain analytically stable in canine urine stored at ambient temperature (20 °C) for up to 168 h prior to −80 °C storage. Observed changes over time were within accepted analytical benchmarks. These findings provide urine-specific validation supporting use of free-catch samples in field-based studies, longitudinal investigations, and biobanking initiatives where immediate processing is not feasible.

Canine urine samples intended for CML and CEL analysis can be held at ambient temperature for up to seven days before freezing at −80 °C, provided that basic handling practices (e.g., prompt cooling after collection, gentle centrifugation to remove cellular debris, and avoidance of repeated freeze–thaw cycles) are followed. Future work should extend stability assessments to additional AGE species and temperature conditions to further develop standardized pre-analytical recommendations for veterinary and comparative biomedical research.

## Data Availability

Data is available in the Supplementary Materials. Any additional questions can be directed to the corresponding author.

## Supplementary Materials

The following supporting information can be downloaded at: https://www.mdpi.com/article/10.3390/ani16060917/s1. File S1.

## Abbreviations

The following abbreviations are used in this manuscript:

AGE	Advanced Glycation End Products
CML	Nε-carboxymethyllysine
CEL	Nε-carboxyethyllysine
LC-MS	Liquid Chromatography—Mass Spectrometry
*m*/*z*	Mass-to-charge ratio
IQR	Interquartile range
